# Increased Expression of Osteopontin in Retinal Degeneration Induced by Blue Light-Emitting Diode Exposure in Mice

**DOI:** 10.3389/fnmol.2016.00058

**Published:** 2016-07-25

**Authors:** Seung Wook Chang, Hyung Il Kim, Gyu Hyun Kim, Su Jin Park, In-Beom Kim

**Affiliations:** ^1^Department of Anatomy, College of Medicine, The Catholic University of KoreaSeoul, Korea; ^2^Gyeongju St. Mary’s Eye ClinicGyeongju, Korea; ^3^Catholic Neuroscience Institute, College of Medicine, The Catholic University of KoreaSeoul, Korea; ^4^Catholic Institute for Applied Anatomy, College of Medicine, The Catholic University of KoreaSeoul, Korea

**Keywords:** osteopontin, retinal degeneration, microglia, inflammation, phagocytosis

## Abstract

Osteopontin (OPN) is a multifunctional adhesive glycoprotein that is implicated in a variety of pro-inflammatory as well as neuroprotective and repair-promoting effects in the brain. As a first step towards understanding the role of OPN in retinal degeneration (RD), we examined changes in OPN expression in a mouse model of RD induced by exposure to a blue light-emitting diode (LED). RD was induced in BALB/c mice by exposure to a blue LED (460 nm) for 2 h. Apoptotic cell death was evaluated by terminal deoxynucleotidyl transferase dUTP nick end labeling (TUNEL) assay. In order to investigate changes in OPN in RD, western blotting and immunohistochemistry were performed. Anti-OPN labeling was compared to that of anti-glial fibrillary acidic protein (GFAP), which is a commonly used marker for retinal injury or stress including inflammation. OPN expression in RD retinas markedly increased at 24 h after exposure, was sustained through 72 h, and subsided at 120 h. Increased OPN expression was observed co-localized with microglial cells in the outer nuclear layer (ONL), outer plexiform layer (OPL), and subretinal space. Expression was restricted to the central retina in which photoreceptor cell death occurred. Interestingly, OPN expression in the ONL/OPL was closely associated with microglia, whereas most of the OPN plaques observed in the subretinal space were not. Immunogold electron microscopy demonstrated that OPN was distributed throughout the cytoplasm of microglia and in nearby fragments of degenerating photoreceptors. In addition, we found that OPN was induced more acutely and with greater region specificity than GFAP. These results indicate that OPN may be a more useful marker for retinal injury or stress, and furthermore act as a microglial pro-inflammatory mediator and a phagocytosis-inducing opsonin in the subretinal space. Taken together, our data suggest that OPN plays an important role in the pathogenesis of RD.

## Introduction

Osteopontin (OPN) is a multifunctional matricellular glycoprotein that contains arginine-glycine-aspartate (RGD) domains. OPN is widely expressed in neurons of the developing and adult brain (Shin et al., 1999; Lee et al., 2001; Iczkiewicz et al., 2004), but its endogenous functions remain unknown. In the pathological brain, OPN is implicated as a mediator of pro-inflammatory effects in a variety of neurodegenerative diseases such as multiple sclerosis (Kim et al., 2004; Hur et al., 2007; Niino and Kikuchi, 2011), Alzheimer’s disease (Wung et al., 2007; Comi et al., 2010; Wirths et al., 2010), Parkinson’s disease (Iczkiewicz et al., 2006; Mattson et al., 2008), and ischemic stroke (Ellison et al., 1998; Wang et al., 1998; Schroeter et al., 2006; Choi et al., 2007). Specifically, OPN is secreted by macrophages, activated microglia, and astrocytes, and is generally thought to promote the inflammatory activation of microglia and macrophage infiltration (Kim et al., 2004; Schroeter et al., 2006; Choi et al., 2007; Wirths et al., 2010). In contrast, there is an accumulating body of evidence indicating that OPN has neuroprotective and repair-promoting effects in the pathogenesis of various brain diseases (Meller et al., 2005; van Velthoven et al., 2011; Shin, 2012). Thus, the role of OPN in the course of brain disease is unclear.

Anatomically and developmentally, the retina is a part of the brain, and retinal diseases share common features with various neurodegenerative diseases. Most notable are the parallels between Alzheimer’s disease and retinal degeneration (RD). That is, both are chronic progressive diseases that are age-related and cause irreversible neuronal cell loss, oxidative stress/metabolic damage, neuroinflammation, and glial reactivity (Ohno-Matsui, 2011; Sivak, 2013). RD is a major cause of vision loss and blindness worldwide and is characterized by the irreversible progressive degeneration of photoreceptors (Papermaster and Windle, 1995; Gregory-Evans and Bhattacharya, 1998; Kim et al., 2016). In the pathogenesis of RD including age-related macular degeneration (AMD), inflammation is strongly implicated (Coleman et al., 2008; Ding et al., 2009; Horie-Inoue and Inoue, 2014; Nita et al., 2014) and microglial cells are believed to play an important role in the initiation and propagation of the inflammatory response and subsequent neuronal cell death (Langmann, 2007; Karlstetter et al., 2010; Madeira et al., 2015). While the expression profiles of various glial inflammatory have been reported in RD, little is known regarding the role of OPN.

In this study, as a first step in understanding the role of OPN in RD, we investigated the spatiotemporal distribution and cellular localization of OPN in a mouse model of RD induced by blue light-emitting diode (LED) exposure.

## Materials and Methods

### Animals

A total of 45 males 7-week-old BALB/c mice were used in this study. Thirty six mice were used to generate RD and the remaining nine mice were used as normal controls. This study was carried out in strict accordance with the recommendations provided in the Guide for the Care and Use of Laboratory Animals of the National Institutes of Health (NIH Publications No. 80-23; revised in 1996). The study protocol was approved by the Institutional Animal Care and Use Committee (IACUC) of the College of Medicine, The Catholic University of Korea (Approval Number: CUMS-2015-0032-02).

### Mouse Model of RD

We employed a blue LED-induced model of RD as described in a previous study (Kim et al., 2016). Briefly, mice were dark-adapted for 24 h and then subjected to pupillary dilation with 0.5% tropicamide and 0.5% phenylephrine hydrochloride ocular drops (Santen, Osaka, Japan) under dim red light (*λ* > 600 nm) for 30 min. Afterwards, mice were exposed to a 2000-lux blue LED (460 ± 10 nm) for 2 h in cages with reflective interiors. Following blue LED exposure, mice were kept in darkness for 24 h and then resumed a 12-h light-dark cycle. At 0, 24, 72, and 120 h after blue LED exposure, electroretinography (ERG) recordings were performed and mice were subsequently sacrificed.

### ERG

All mice were dark-adapted for 1 h before ERG recording. Then, under dim red light (*λ* > 600 nm), animals were anesthetized with 8% chloral hydrate (0.5 mL/kg, i.p.) and placed on a heating pad in order to maintain body temperature at 35–36°C. Mice were fixed to the top of a stage with the eyes facing a light source at a 20-cm distance. Corneas were coated with hydroxypropyl methylcellulose gel and covered by gold ring contact electrodes. Stimuli were brief white flashes of light delivered via a Ganzfeld stimulator (UTAS-3000; LKC Technologies, Gaithersburg, MD, USA). Signals were amplified and filtered through a digital band-pass filter ranging from 5 to 100 Hz in order to elicit a- and b-waves. Scotopic ERGs obtained for flash intensities ranging from −2.7 to 0.9 log (cd·s)/m^2^ were recorded at three stimulus levels. Each recording was the average of all responses obtained within a 15-s interstimulus interval.

### Hematoxylin and Eosin (H&E) Staining

The anterior segments of the eyes were removed and the eyecups were fixed by immersion in 4% paraformaldehyde in 0.1 M phosphate buffer (PB: pH 7.4) for 2 h. Afterwards, tissues were rinsed in PB, transferred to a 30% sucrose solution in PB, infiltrated overnight, and embedded the next day in a supporting medium for frozen tissue specimens (Tissue-Tek OCT compound; Sakura, Alphen aan den Rijn, Netherlands). Vertical tissue sections (7 μm) were cut on a cryostat at −25°C, stored at −20°C, and subsequently stained with H&E.

### Terminal Deoxynucleotidyl Transferase dUTP Nick End Labeling (TUNEL) Assay

Terminal deoxynucleotidyl transferase dUTP nick end labeling (TUNEL) assays were performed in 7-μm cryosections according to manufacturer specifications (*In Situ* Cell Death Detection kit; Roche Biochemicals, Mannheim, Germany). Briefly, sections were dewaxed and washed in 0.01 M phosphate buffered saline (PBS) for 30 min, incubated with a permeabilization solution (0.1% Triton-100, 0.1% sodium citrate) for 2 min on ice, and subsequently incubated with terminal deoxynucleotidyl transferase enzyme at 37°C for 1 h. Cell nuclei were counterstained with 4′,6-diamidino-2′-phenylindole (DAPI; dilution, 1:1000; Invitrogen, Eugene, OR, USA). Labeling was visualized on a Zeiss LSM 510 Meta confocal microscope (Carl Zeiss Co., Ltd., Oberkochen, Germany).

### Western Blotting

Western blot analyses were performed on extracts of the eyecups, which were homogenized in ice-cold lysis buffer (1% sodium dodecyl sulfate, 1.0 mM sodium orthovanadate, 10 mM Tris, pH 7.4). Aliquots of tissue each containing 50 μg of total protein were heated at 100°C for 10 min with an equivalent volume of 2× sample buffer and loaded onto 10% polyacrylamide gels. Proteins were electrophoresed and subsequently blotted onto a polyvinylidene fluoride membrane. The membrane was blocked with 5% nonfat dry milk dissolved in 0.01 M PBS (pH 7.4) containing 0.05% Tween-20 for 1 h at room temperature. The membrane was then incubated for 15 h at 4°C with goat anti-OPN polyclonal antibody (1:2000; R&D Systems, Minneapolis, MN, USA) in blocking solution. The membrane was rinsed three times with PBS containing 0.05% Tween-20 (10 min per wash), and then incubated with peroxidase-conjugated donkey anti-goat IgG antibody (1:1000; Jackson ImmunoResearch, West Grove, PA, USA) for 2 h at room temperature. Blots were developed using the Enhanced Chemiluminescence Detection Kit (Amersham, Arlington Heights, IL, USA) and densitometry was performed using the Eagle Eye TMII Still Video System (Stratagene, La Jolla, CA, USA). Data are represented as the means ± standard deviation (SD). Statistical significance was assessed with Student’s *t*-test for comparisons between normal control and blue LED-induced RD groups; *P* < 0.05 was considered to indicate statistical significance.

### Immunohistochemistry

Cryosections were washed three times with PB and blocked in 10% normal donkey serum in PB for 1 h at room temperature. Sections were then incubated with goat anti-OPN polyclonal antibody (1:1000), rabbit polyclonal anti-ionized calcium binding adaptor molecule 1 (Iba1) antibody (1:1000; Wako Pure Chemical Industries, Osaka, Japan), or rabbit anti-glial fibrillary acidic protein (GFAP) polyclonal antibody (1:1000; Chemicon, Temecula, CA, USA) in PB for 5 h at room temperature. Sections were subsequently washed in PB and incubated with Alexa 488-conjugated donkey anti-goat (1:1000; Molecular Probes, Eugene, OR, USA) or Cy3-conjugated anti-rabbit IgG (1:1000) for 2 h. Cell nuclei were counterstained with DAPI. Fluorescent specimens were mounted with Vectashield mounting media (Vector Laboratories, Burlingame, CA, USA) and imaged using a confocal microscope (Carl Zeiss Co., Ltd., Jena, Germany).

### Immunogold Electron Microscopy

For immunogold electron microscopy, segments of eyecup tissue harvested from blue LED-induced RD mice were fixed by immersion in 4% paraformaldehyde in PB for 2 h. Vibratome sections (50 μm) were incubated with anti-OPN antibody as described above. Sections were then incubated with a bridge antibody (mouse anti-goat IgG), washed thoroughly, and incubated with a nanogold particle-conjugated (1 nm) anti-mouse secondary antibody (1:100; Nanoprobes, Stony Brook, NY, USA) for 2 h. Silver enhancement was performed using the HQ silver enhancement kit (Nanoprobes) for 3 min. Next, sections were postfixed in 1% glutaraldehyde in PB and subsequently 1% osmium tetroxide in PB for 30 min each and dehydrated in a graded series of ethanol. Sections were finally embedded in Epon 812 resin and ultrathin sections (70–90 nm) were cut on an ultramicrotome, stained with 1% uranyl acetate, and visuallized with an electron microscope (JEM 1010, JEOL, Tokyo, Japan).

## Results

### Blue LED-Induced RD

**Figure 1:**
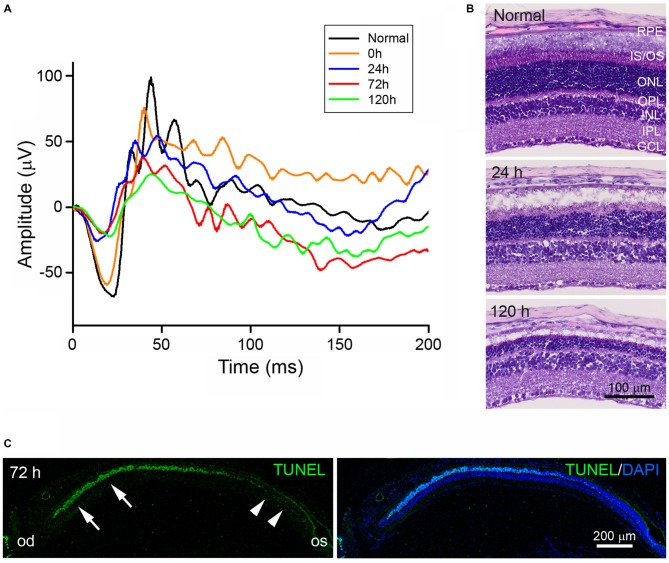
**Characterization of blue light-emitting diode (LED)-induced retinal degeneration (RD) in mice. (A)** Representative scotopic electroretinography (ERG) responses in unexposed control (black) and blue LED-induced retinas at 0 (orange), 24 (blue), 72 (red), and 120 h (green). All ERG components showed progressive reductions in a time-dependent manner. **(B)** Hematoxylin and Eosin (H&E) staining of representative retinal tissue sections. Consistent with ERG findings, retinal thickness decreased in a time-dependent manner. Prominent decreases were observed in the outer nuclear layer (ONL). GCL, ganglion cell layer; INL, inner nuclear layer; IPL, inner plexiform layer; IS/OS, inner segment and outer segment; OPL, outer plexiform layer; RPE, retinal pigment epithelium. **(C)** Terminal deoxynucleotidyl transferase dutp nick end labeling (TUNEL) staining on a vertical section of eyecup taken from an RD mouse at 72 h after blue LED exposure. Numerous TUNEL-positive photoreceptors were observed in the ONL of the central retina (arrows) near the optic disc (od), while few TUNEL-positive cells were observed in the peripheral retina (arrowheads) near the ora serrata (os). 4′,6-diamidino-2′-phenylindole (DAPI) staining was used to label the nuclei of retinal cells.

To confirm and characterize blue LED-induced RD, scotopic ERG recordings were measured (Figure 1A). The amplitudes of both a- and b-waves of ERG responses were reduced immediately after (0 h) blue LED exposure as compared to normal control amplitudes. At 120 h after blue LED exposure, the amplitudes of a- and b-waves were 27% and 29% of normal control amplitudes, respectively. Consistent with scotopic ERG findings, H&E staining demonstrated a progressive reduction in retinal thickness over time that was mainly due to the loss of photoreceptors in the outer nuclear layer (ONL; Figure 1B): more than 15 layers of photoreceptors were observed in the ONL of the central retina in normal control mice, while only 3–5 layers were observed in RD mice at 120 h after blue LED exposure. Additionally, in RD mice, numerous TUNEL-positive photoreceptors were observed in the central retina and few TUNEL-positive cells were observed in the peripheral retina (Figure 1C). These findings demonstrate that blue LED exposure induces a state of RD primarily in the central retina.

### Temporal and Spatial Profiles of OPN Expression in RD Retinas

**Figure 2:**
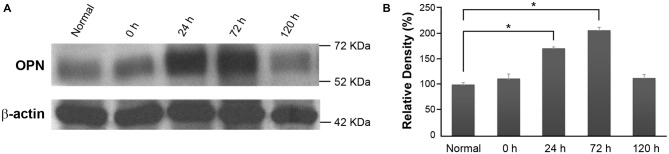
**Osteopontin (OPN) expression in blue LED-induced RD retinas. (A)** Representative western blot. A representative 66-kDa band was identified with anti-OPN labeling and thus recognized as OPN. **(B)** Densitometric analysis of the 66 kDa OPN band. Results are expressed as percentages (%) relative to normal control retina values. Data represent the mean ± standard deviation (SD) for four mice in each group. *P < 0.05.

In order to investigate changes in OPN expression over time in RD retinas, total protein extracts from the eyecups of normal control and blue LED-exposed mice were subjected to western blotting (Figure 2A). A representative 66-kDa band was identified as consistent with the size of OPN and quantitatively analyzed by densitometry (Figure 2B). OPN expression was significantly increased in RD retinas at 24 h and 72 h after blue LED exposure (*P* < 0.05) compared to that in normal control retinas.

**Figure 3:**
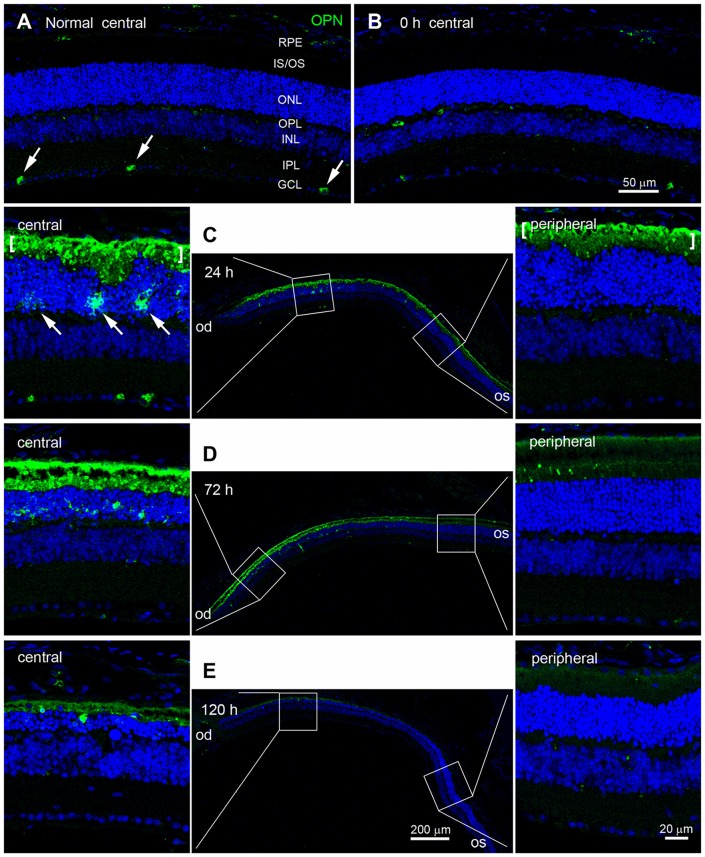
**Temporal and spatial profiles of OPN expression in blue LED-induced RD retinas.** Confocal micrographs taken from vertical sections of blue LED-induced RD eyecups processed for OPN immunoreactivity. **(A)** A representative normal control retina. Several ganglion cells (arrows) in the GCL were weakly labeled with OPN. INL, inner nuclear layer; IPL, inner plexiform layer; IS/OS, inner segment and outer segment; ONL, outer nuclear layer; OPL, outer plexiform layer; RPE, retinal pigment epithelium. **(B)** A representative RD retina immediately after blue LED exposure (0 h). OPN expression was similar to that in the unexposed RD control. **(C)** A representative RD retina at 24 h after blue LED exposure. The central and mid-peripheral retina are magnified. Strong OPN immunoreactivity was observed in cells of the ONL (arrows) and in the subretinal space (bracket) in the central retina, but not in the peripheral retina. **(D)** A representative RD retina at 72 h after blue LED exposure. OPN expression was similar to that in the RD retina at 24 h after blue LED exposure; however, a relatively larger number of OPN-labeled cells were observed in the ONL of the central retina at this time point. OPN immunoreactivity in the subretinal space of the peripheral retina was negligible. **(E)** A representative RD retina at 120 h after blue LED exposure. OPN immunoreactivities in the ONL and subretinal space of the central retina were markedly decreased.

Next, we used immunohistochemistry to examine changes in the pattern of OPN expression in blue LED-exposed retinas. Consistent with previous reports (Ju et al., 2000; Moon et al., 2005), OPN was expressed in a subset of ganglion cells in the ganglion cell layer (GCL) of normal control retinas (Figure 3A). Immediately after blue LED exposure (0 h), the pattern of OPN expression in AD mice was not significantly different from that in normal control mice (Figure 3B). However, at 24 h after blue LED exposure, OPN expression was dramatically increased (Figure 3C). Strong OPN expression was observed in the subretinal space and in cells of the ONL (arrows in Figure 3C). Interestingly, OPN-positive cells were only observed in the central region of the retina, confirming our findings regarding the selective induction of RD in the central retina. Changes in OPN expression persisted until 72 h in RD mice, but were quantitatively lesser than those observed at 24 h, particularly in the subretinal space (Figure 3D). Spatial differences (central vs. peripheral) in OPN expression at 72 h were also consistent with those observed at 24 h in RD mice (Figure 3C). At 120 h after blue LED exposure, OPN expression had subsided, particularly in the subretinal space (Figure 3E).

It should furthermore be noted that the labeling patterns of the ONL/outer plexiform layer (OPL) and subretinal space were different. That is, OPN-labeling in the ONL and OPL appeared diffuse, which was suggestive of cytoplasmic localization within the cell, whereas labeling in the subretinal space appeared as small plaques (Figures 3C–E).

### Cellular Localization of OPN in RD Retinas

**Figure 4:**
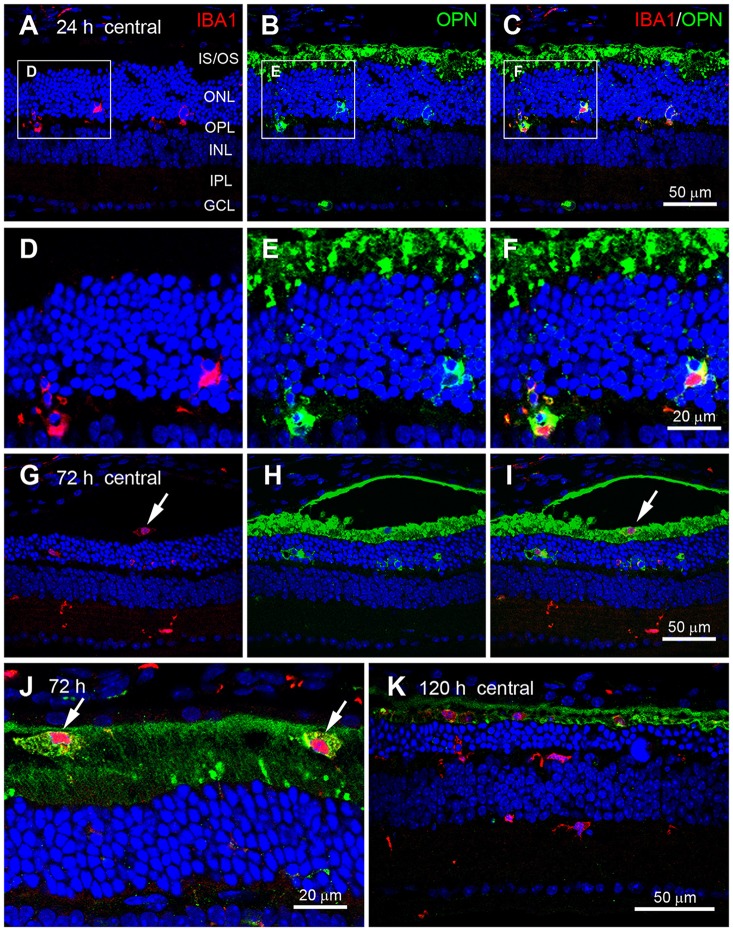
**Cellular localization of OPN in blue LED-induced RD retinas.** Confocal micrographs taken from vertical sections of blue LED-induced RD retinas processed for OPN (green) and Iba1 (red) immunoreactivity. **(A–F)** A representative RD retina at 72 h after blue LED exposure. OPN was observed in Iba1-labeled microglia (arrows) in the ONL and OPL. INL, inner nuclear layer; IPL, inner plexiform layer; IS/OS, inner segment and outer segment; ONL, outer nuclear layer; OPL, outer plexiform layer; RPE, retinal pigment epithelium. A region including two OPN-labeled microglia in **(A–C)** are magnified in **(D–F)**, respectively. OPN was localized in the processes of Iba1-labeled microglial cells. **(G–J)** A representative RD retina at 72 h after blue LED exposure. Iba1-labeled cells were observed in the INL, IPL, and subretinal space. OPN was observed in Iba1-labeled microglia of the ONL and microglia/macrophages of the subretinal space (arrow), while OPN expression was not observed in Iba1-labeled microglia of the INL or IPL. Two microglia/macrophages (arrows) showing Iba1 and OPN co-labeling are magnified in **(J)**. **(K)** A representative RD retina at 120 h after blue LED exposure. Several Iba1-labeled microglia/macrophages were still observed in the ONL and subretinal space, while minimal OPN-labeling was observed in the ONL and subretinal space.

OPN is mainly secreted by activated microglia during pathological conditions in the brain (Kim et al., 2004; Schroeter et al., 2006; Choi et al., 2007) and retina (Hikita et al., 2006; Chidlow et al., 2008). Thus, we examined whether OPN expression co-localized with Iba1, a microglial cell marker (Imai et al., 1996; Hikita et al., 2006), in blue LED-induced RD retinas. At 24 h after blue LED exposure (Figures 4A–C), OPN was observed co-localized with Iba1 in the ONL and OPL (Figures 4D–F), indicating OPN expression in microglial processes. At 72 h after blue LED exposure (Figures 4G–I), Iba1 was frequently detected in the inner nuclear layer (INL) and inner plexiform layer (IPL), as well as in the ONL and OPL. Interestingly, OPN co-localized with Iba1 in the ONL and OPL, but not in the INL or IPL. In addition, OPN co-localized with Iba1 in the subretinal space (Figures 4I,J). At a higher magnification, differences in the labeling patterns of OPN were once more noted in the ONL/OPL vs. subretinal space: diffuse labeling was observed in the cytoplasm of putative microglia in the ONL and OPL (Figure 4F), whereas small plaques of OPN were observed in putative microglia of the subretinal space (Figure 4J). At 120 h after blue LED exposure, OPN and Iba-1 co-localization was almost negligible due to decreases in OPN immunoreactivity, and minor co-localization was observed in the subretinal space (Figure 4K).

### Subcellular Localization of OPN in RD Retinas

**Figure 5:**
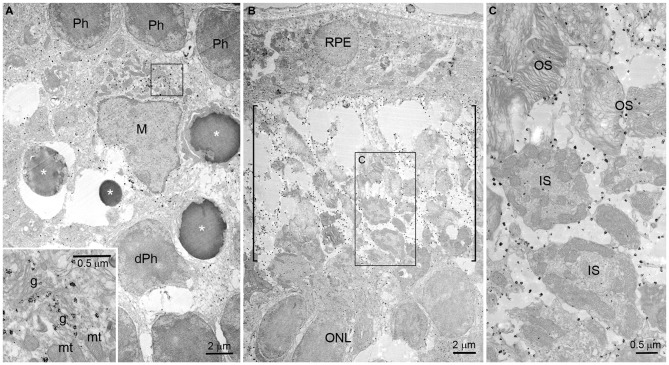
**Immunogold electron microscopy of OPN in blue LED-induced RD retinas.** Electron micrographs taken from vertical sections of an RD retina at 72 h after blue LED exposure that was processed for OPN immunoreactivity. **(A)** OPN localization in the ONL. OPN labeled with immunogold was observed in the cytoplasm of microglia (M) in the ONL. Photoreceptors (Ph) ongoing apoptosis (asterisks) or with degenerative changes (dPh) were frequently observed nearby. A portion of microglial cytoplasm (rectangle) is magnified in the *inset*. In the *inset*, immunogold is observed associated with vesicular structures proximal to Golgi complexes (g) but not with mitochondria (mt). **(B,C)** OPN localization in the subretinal space. Large numbers of immunogold particles were present in subretinal space (bracket). In a higher magnification view **(C)** of the rectangular region **(B)**, immunogold is observed associated with the membranes of fragmented or degenerating outer segments (OS) packed with membranous discs, and inner segments (IS) containing numerous mitochondria. RPE, retinal pigment epithelium.

Using immunogold electron microscopy, we further investigated the localization of OPN in blue LED-induced RD retinas. At 72 h after blue LED exposure, OPN labeled with immunogold was observed in microglia and the cytosolic fragments of the photoreceptors ongoing apoptosis or degeneration (Figure 5). In microglia, labeling was widely distributed throughout the cytoplasm (Figure 5A). Occasionally, labeled vesicular structures were closely associated with Golgi complexes but never with mitochondria (*inset* in Figure 5A). Additionally, large numbers of immunogold particles were present in the subretinal space (Figure 5B), and exclusively associated with fragmented or degenerating photoreceptor outer segments (OS; packed with membranous discs) and inner segments (IS; containing numerous mitochondria; Figure 5C).

### Expression Pattern of GFAP in RD Retinas

**Figure 6:**
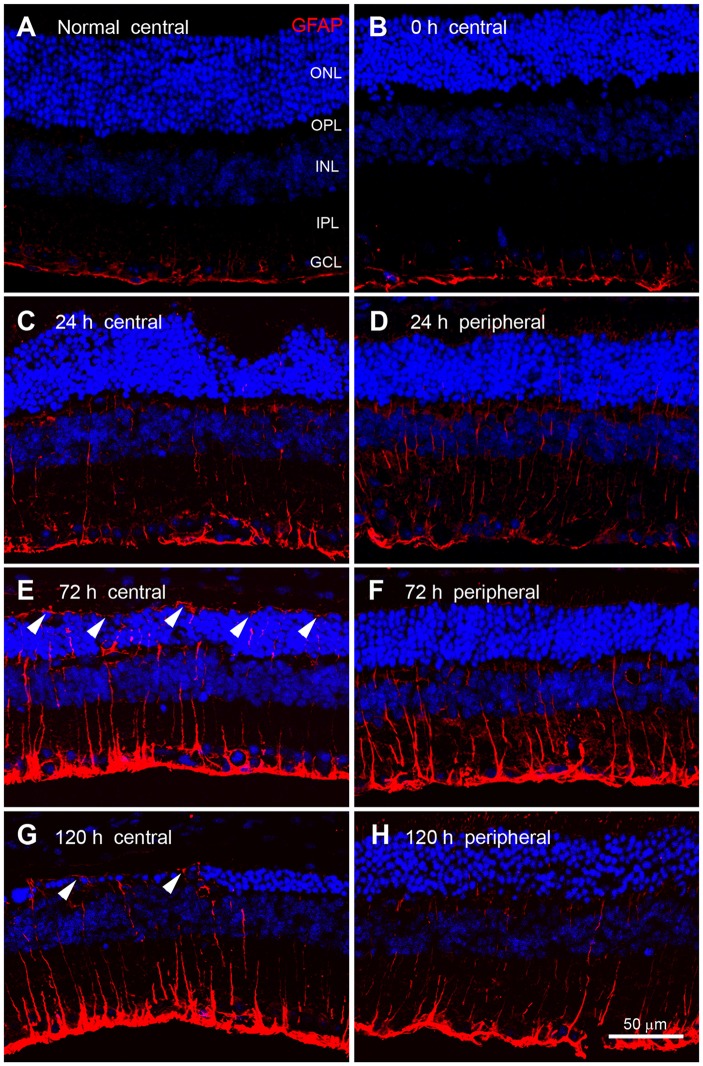
**Glial fibrillary acidic protein (GFAP) immunoreactivity in blue LED-induced RD retinas.** Confocal micrographs taken from vertical sections of the central (left panel) and peripheral (right panel) retina processed for GFAP (red) immunoreactivity in normal **(A)** and blue LED-induced RD mice **(B–E)**. **(A)** A representative normal control retina. GFAP was expressed in astrocytes and Müller cell endfeet in the GCL. **(B)** A representative RD retina immediately after blue LED exposure (0 h). Expression of GFAP was similar to that in the normal control. **(C,D)** A representative RD retina at 24 h after blue LED exposure. GFAP immunoreactivity was slightly increased relative to 0 h. Thin GFAP-labeled Müller cell processes were found in the INL and the IPL. There were no differences in GFAP expression between the central **(C)** and peripheral **(D)** retina at this time point. **(E,F)** A representative RD retina at 72 h after blue LED exposure. GFAP immunoreactivity was increased in both the central **(E)** and peripheral **(F)** retina, particularly in the ONL of the central retina, where GFAP-labeled Müller cell processes (arrowheads) extended to the outer limiting membrane. **(G,H)** A representative RD retina at 120 h after blue LED exposure. GFAP immunoreactivity was decreased in both the central **(G)** and peripheral **(H)** retina, but GFAP-labeling (arrowheads) persisted in the outer limiting membrane.

We next compared the expression patterns of OPN and GFAP, a representative marker of retinal injury or stress (Kim et al., 1998; Jeong et al., 2011; Paik et al., 2012), in blue LED-induced RD retinas. In normal control retinas and immediately after blue LED exposure (0 h), GFAP immunoreactivity was observed in the endfeet of Müller cells and astrocytes of the GCL (Figures 6A,B). At 24 h after blue LED exposure, GFAP immunoreactivity was slightly increased in the central (Figure 6C) and peripheral retina (Figure 6D), and thin Müller cell processes containing GFAP were observed in the INL and IPL. At 72 h after blue LED exposure, GFAP expression was increased in both the central (Figure 6E) and peripheral retina (Figure 6F). GFAP-labeled Müller cell processes had extended to the outer limiting membrane, penetrating the ONL and OPL, and processes in the INL and IPL were observed to be thicker than those observed at 24 h. At 120 h after blue LED exposure, GFAP expression had just slightly subsided, but remained above baseline (Figures 6G,H).

## Discussion

AMD as a representative of RD is the leading cause of vision loss and blindness in the worldwide elderly population (Resnikoff et al., 2004; Fletcher, 2010). Inflammation is strongly implicated in the development and progression of AMD (Coleman et al., 2008; Ding et al., 2009; Horie-Inoue and Inoue, 2014; Nita et al., 2014). Microglia play an important role in the initiation and propagation of the inflammatory response, which is thought to lead to the excessive generation of inflammatory mediators and subsequent neuronal cell death in RD (Langmann, 2007; Karlstetter et al., 2010; Madeira et al., 2015). In clinical practice, intravitreal injection of steroids or antibody against tumor necrosis factor alpha (TNF-α), a key microglial inflammatory mediator, is effective in AMD patients (Theodossiadis et al., 2009; Becerra et al., 2011). In this study, we examined changes in the expression of OPN, another pro-inflammatory mediator known to be secreted by microglia, in a model of RD induced by blue LED exposure. Two interesting features of OPN expression emerged from our findings: a specific spatial distribution, and a distinct temporal profile.

Increased OPN expression following blue LED exposure-induced RD was primarily observed in the central retina (Figure 3), where massive photoreceptor cell death occurred. Moreover, OPN expression was restricted to the ONL, OPL, and subretinal space (Figure 3), where dying photoreceptor cell bodies, axon terminals, and the IS/OS junctional layer are located, respectively. In these retinal regions, OPN was localized to microglia and degenerating photoreceptor components (Figure 4). At the subcellular level, OPN was distributed throughout the cytoplasm of microglia and in nearby fragments of degenerating photoreceptors (Figure 5). In addition, OPN-labeled vesicles associated with Golgi complexes were found within microglia (*inset* in Figure 5). Given that OPN is produced and secreted by activated microglia, and acts as a pro-inflammatory mediator in a variety of neurodegenerative diseases (Kim et al., 2004; Schroeter et al., 2006; Choi et al., 2007; Wirths et al., 2010), our findings suggest that OPN plays an important role in inflammation proximal to the primary injury site and is thus useful as a marker of retinal injury. This suggestion is corroborated by two previous reports which demonstrated in an excitotoxic/ischemic model of glaucoma that increased OPN expression is restricted to the inner retina, particularly the IPL and the GCL (Chidlow et al., 2008), while in an autoimmune uveitis model OPN expression was dominant in the outer retina, particularly the OPL (Hikita et al., 2006).

In addition, we observed that OPN expression co-localized with Iba1 expression and was abruptly increased in RD retinas at 24 h after blue LED exposure, but subsided by 120 h (Figures 2, 3). In contrast, GFAP expression tended to increase slowly but also subsided by 120 h (Figure 6). These different temporal profiles might reflect the different roles of retinal glia. There are two primary types of glial cells in the mammalian retina: Müller cells and microglia. Müller cells and astrocytes in the retina are mainly involved in gliosis tissue repair and remodeling over time (Chen and Swanson, 2003; Reichenbach and Bringmann, 2010). Müller cells express GFAP in response to injury or conditions such as inflammation, such that GFAP is considered to be a representative marker for retinal injury or stress (Kim et al., 1998; Lewis and Fisher, 2003; Jeong et al., 2011; Paik et al., 2012). An interesting feature of GFAP expression is that expression initiates at the endfeet of Müller cells, regardless of the type of retinal injury; this effect has been documented in glaucomatous conditions induced by axotomy (Scherer and Schnitzer, 1991), ischemia-reperfusion injury due to increased intraocular pressure (Kim et al., 1998; Zhang et al., 2009), cauterization of the episcleral veins (Kanamori et al., 2005; Xue et al., 2006), N-methyl-D-aspartate or kainate excitotoxicity (Moncaster et al., 2002; Chang et al., 2007), and various RD conditions induced by N-methyl-N-nitrosourea (Jeong et al., 2011), light (Torbidoni et al., 2006; Kim et al., 2016), or retinal detachment (Lewis and Fisher, 2003; Luna et al., 2010). In contrast, microglia play a pro-inflammatory role in pathological contexts by producing inflammatory cytokines and reactive oxygen species in an effort to eliminate pathogens and cellular debris; however, this function leads to neuronal cell death in the acute phases of injury (Cherry et al., 2014; Madeira et al., 2015). Thus, OPN may be more relevant in acute injury states. This temporal profile is consistent with findings from a transient forebrain ischemia model, where OPN mRNA was upregulated in microglia within 3–24 h of injury, peaked at 3 days post-injury, and decreased by 7 days post-injury (Choi et al., 2007). Taken together, OPN appears to play a role akin to heat shock proteins in the immediate stress response to pathological conditions, and thus, can be a useful marker for retinal stress and injury.

A growing body of evidence indicates that OPN may also act as a phagocytosis-inducing opsonin. OPN facilitates mineralized particle uptake by macrophages *in vitro* (Pedraza et al., 2008) and, in a transient focal ischemia model, OPN was observed to accumulate on the surface of cell fragments phagocytosed by microglia (Shin et al., 2011). Moreover, OPN has been observed in association with calcium deposits prior to scavenging (Shin et al., 2012). Very recently, OPN expression was detected in the mitochondria of degenerating striatal neurons, and OPN-labeled mitochondria fragments were observed in activated brain macrophages in a rat model of Huntington’s disease (Kim et al., 2015). In the present study, OPN in the subretinal space (regardless of whether or not it was co-localized with Iba1 in microglia) appeared in plaques of various sizes (Figure 4). The subretinal space contains the IS/OS junctional photoreceptor layer, and accordingly large amounts of mitochondria and membranous discs. In blue LED exposure-induced RD, we have previously reported the observation of degenerating mitochondria and phagocytosing microglia (Kim et al., 2016). In the present study, electron microscopy demonstrated that OPN was localized in the membranes of degenerating or fragmented IS and OS where mitochondria and membranous discs were packed with, respectively, but not inside of mitochondria (Figure 5). Taken together, these findings indicate that the observation of OPN plaques in the subretinal space represent localization in fragmented IS/OS and those engulfed by microglia. Thus, OPN has a potential role as a phagocytosis-inducing opsonin in RD.

The present study demonstrates in a mouse model of RD induced by blue LED exposure that OPN expression acutely increases within 24 h following injury, is sustained through 72 h, and subsides by 120 h. Increases in OPN expression were selectively observed in the central retina, the primary site of photoreceptor apoptosis, and particularly restricted to the ONL, OPL, and subretinal space. The observed spatiotemporal pattern of expression indicates that OPN may be a more suitable marker for retinal stress or injury than GFAP, which demonstrated a slower induction of expression that was restricted to the endfeet of Müller cells of the GCL in our study. In addition, the identification of OPN expression in microglia of the ONL, OPL, and subretinal space and in fragments of degenerating photoreceptors in the subretinal space indicate a potential role for OPN as a pro-inflammatory mediator and a phagocytosis-inducing opsonin in RD.

## Author Contributions

SWC and I-BK conceived and designed the experiment. SWC and GHK set up the RD mouse model, performed ERG, TUNEL staining, and immunohistochemistry. HIK performed western blotting, densitometry, and immunogold electron microscopy. SJP and HIK made significant contributions to taking confocal images and analyzing the ERG data, respectively. SWC, HIK, and I-BK wrote the article. All authors read and approved the final manuscript.

## Funding

This work was supported by the Basic Science Research Program through the National Research Foundation (NRF) of Korea funded by the Ministry of Education, Science, and Technology (2013R1A2A2A01014070).

## Conflict of Interest Statement

The authors declare that the research was conducted in the absence of any commercial or financial relationships that could be construed as a potential conflict of interest.
